# Integrating domain similarity to improve protein complexes identification in TAP-MS data

**DOI:** 10.1186/1477-5956-11-S1-S2

**Published:** 2013-11-07

**Authors:** Bingjing Cai, Haiying Wang, Huiru Zheng, Hui Wang

**Affiliations:** 1 School of Computing and Mathematics, Computer Sciences Research Institute, University of Ulster, N. Ireland, BT37 0QB, UK

## Abstract

**Background:**

Detecting protein complexes in protein-protein interaction (PPI) networks plays an important role in improving our understanding of the dynamic of cellular organisation. However, protein interaction data generated by high-throughput experiments such as yeast-two-hybrid (Y2H) and tandem affinity-purification/mass-spectrometry (TAP-MS) are characterised by the presence of a significant number of false positives and false negatives. In recent years there has been a growing trend to incorporate diverse domain knowledge to support large-scale analysis of PPI networks.

**Methods:**

This paper presents a new algorithm, by incorporating Gene Ontology (GO) based semantic similarities, to detect protein complexes from PPI networks generated by TAP-MS. By taking co-complex relations in TAP-MS data into account, TAP-MS PPI networks are modelled as bipartite graph, where bait proteins consist of one set of nodes and prey proteins are on the other. Similarities between pairs of bait proteins are computed by considering both the topological features and GO-driven semantic similarities. Bait proteins are then grouped in to sets of clusters based on their pair-wise similarities to produce a set of 'seed' clusters. An expansion process is applied to each 'seed' cluster to recruit prey proteins which are significantly associated with the same set of bait proteins. Thus, completely identified protein complexes are then obtained.

**Results:**

The proposed algorithm has been applied to real TAP-MS PPI networks. Fifteen quality measures have been employed to evaluate the quality of generated protein complexes. Experimental results show that the proposed algorithm has greatly improved the accuracy of identifying complexes and outperformed several state-of-the-art clustering algorithms. Moreover, by incorporating semantic similarity, the proposed algorithm is more robust to noises in the networks.

## Background

Protein complexes, in which multiple proteins physically interact with each other, are essential to organization and functions of cellular machines [1,2]. As the advance of experimental and computational technologies, an immense amount of protein-protein interactions (PPIs) have been detected [3-8], which can be represented as in the form of networks. Thus, the accurate identification of protein complexes from such large-scale networks of PPIs becomes a challenge.

Yeast-two-hybrid (Y2H) and tandem affinity-purification/mass-spectrometry (TAP-MS) are two types of high-throughput experimental techniques which have been widely applied to detect PPIs. Y2H identifies physically pair-wise PPIs [3,4] while TAP-MS detects co-complex relations of complexes by purifying proteins (called prey) that are associated with tagged proteins (called bait) [5,6,8].

A network of PPIs is generally represented as an undirected simple graph where proteins correspond to nodes and pair-wise interactions correspond to edges. Graph-based clustering algorithms are an effective approach to identify protein complexes. In 2000, Markov Clustering Algorithm (MCL) [9] was proposed for identifying complexes from protein interaction networks by simulating random walks on the graph. During the clustering process, an inflation parameter is applied to enhance the contrast between regions of dense and sparse connections in the graph. The process converges towards a partition of the graph, with a set of sub-graphs of high density. In 2003, Bader and Hogue [10] represented PPI networks using their proposed 'Spoke' model and the 'Matrix' model, and applied the Molecular Complex Detection (MCODE) algorithm to detecting protein complexes from the two models. MCODE identifies sets of nodes in which are highly connected, based on the density of neighbours of nodes in the network. In 2006, Brohée and Helden [11] carried out an evaluation on the performance of four clustering algorithms in detecting protein complexes, including MCL and MCODE. Evaluation results showed that comparing to other algorithms, MCL demonstrated its robustness in the context of adding noises to the graph. In 2006, CFinder [12] was proposed to detect overlapping clusters. It explores clusters which are composed of numbers of *k*-cliques where two adjacent *k*-cliques share *k*-1 nodes. Later, a random walk based clustering algorithm, Repeated Random Walks (RRW) [13], was proposed to identify overlapping protein complexes in PPI networks and experimental results demonstrated that RRW obtained clusters with higher precision than MCL [12]. A novel core-attachment based algorithm, COACH, was proposed in 2009 [14]. COACH detects protein complexes with highly-dense structure and explores the "core-attachment" organization inside protein complexes. Experimental results [14] showed that COACH achieved better performance than several existing clustering algorithms.

The algorithms introduced above treat PPIs from TAP-MS data as binary. In recent years, several researchers take advantage of non-binary nature of TAP-MS data, the co-complex relations between bait proteins and prey proteins, to identify protein complexes. In 2005, Scholtens et al. [15] modelled TAP-MS data as a directed graph where edges link from bait proteins to prey proteins, and then applied Local Modelling algorithm [15] to this directed network to search for dense sub-networks in which all pairs of proteins should be connected. Results showed that predicted complexes from the Local Modelling algorithm mapped well to curated protein complexes. Another example of detecting protein complexes by building a non-binary model for TAP-MS data is a novel algorithm called CODEC [16] proposed in 2011. CODEC constructs a bipartite graph to represent TAP-MS data, where one set consisting only of bait proteins while the other set consisting of prey proteins. Edges only link nodes in the two opposite sets. CODEC identifies dense bipartite sub-graphs. Experimental results [16] showed the CODEC outperformed other algorithms with higher precision. In 2012, a new bipartite graph based clustering algorithm (BGCA) was developed to identify protein complexes from TAP-MS PPI networks [17]. Experimental results demonstrated that, the BGCA algorithm achieved significant improvement in identifying protein complexes from TAP-MS data. Greater precision and better accuracy have been achieved and the identified complexes were demonstrated to match well with existing curated protein complexes.

Algorithms introduced above have been developed based on topological features of PPI networks. However, due to experimental limitations, there exist false positives and false negatives in PPIs. Besides physically interacting pair-wise relationships between proteins, semantic similarity describes another type of relationship between pairs of proteins by measuring closeness between the two proteins which is based on estimates of ontology-based functional similarity [18,19]. The Gene Ontology (GO) [20] is the main focus of investigation of semantic similarity in molecular biology [18]. Many measures [19,21-23] for computing semantic similarities have been proposed by using annotations from the three GO hierarchies [20] - Molecular Function (MF), Biological Process (BP), and Cellular Component (CC). It has been confirmed that GO-driven similarity among genes is a relevant indicator of functional interaction in the investigation of assessment and evaluation of semantic similarity [18]. Results in the study [24] also demonstrated that there is a significant correlation between the semantic similarity of pair-wise proteins and their co-complex membership. It is showed that semantic similarity assists validating the results which are obtained from biomedical studies, such as gene clustering and gene expression data analysis [19]. Therefore, in the paper, it is assumed that incorporating semantic similarity into clustering process can improve the accuracy of identifying protein complexes.

Cai et. al [17] demonstrated that good performance of BGCA in detection of protein complexes in TAP-MS PPI network. BGCA identifies protein complexes relying on topological similarity between pairs of bait proteins which is calculated based on the number of commonly shared prey proteins. This paper proposes a new algorithm, which is extended from BGCA, to detect protein complexes from TAP-MS data by integrating semantic similarity. Similarity between pairs of bait proteins is obtained by combining topology-based similarity and GO-driven semantic similarity. An agglomerate hierarchical clustering approach is applied to group bait proteins in to clusters which demonstrate greater similarity among proteins in the same cluster than in different cluster. Thus, a set of 'seed' clusters composed of bait proteins is produced. Starting from these 'seed' clusters, a greedily expansion process is developed to recruit prey proteins which are significantly associated with the same set of bait proteins. After expanding from each seed cluster, a final set of protein complexes is outputted. Experimental results demonstrate that, by integrating semantic similarity, not only the accuracy of detection of proteins complexes has been improved, but also the robustness of the algorithm. This paper is an extension from the conference paper [25]. Based on the paper [25], this paper employs more statistical measures to evaluate quality of clustering results of the proposed method. Moreover, the statistical significance of the clustering results of the proposed algorithm is examined by investigating the estimates of random expectation of correct grouping by randomising predicted complexes sets, and the robustness of the proposed algorithm is also investigated.

The organization of the paper is shown as below. We first introduce the methodology of our proposed algorithm followed the presentation and discussion of experimental results. The propose algorithm is applied to two real world TAP-MS PPI networks. Several statistical metrics are employed to assess the quality of clustering. Statistical significance of clustering results and the robustness of the proposed algorithm to the false negatives and false positives are also evaluated. Finally, the conclusion and future work is presented.

## Methods

Our proposed algorithm is developed from BGCA, which was proposed to detect protein complexes by modelling TAP-MS PPI networks as bipartite graph [17]. The algorithm lies on the assumption that, as TAP-MS experiment directly detects complex membership by purifying prey proteins which are co-associated with tagged bait proteins [5,6], a protein complex is institutively composed of a set of bait proteins along with a set of prey proteins that are significantly associated with the same set of bait proteins. Therefore, the core idea in the proposed algorithm is firstly to detect seed clusters composed of bait proteins and then greedily expand from these seed clusters to obtain final clusters. We obtain 'seed' clusters by grouping bait proteins based on their similarities. In this paper, we incorporate GO-based semantic similarity with the topology-based similarity. The proposed algorithm has the same process as BGCA [17], the difference lies in the calculation of pair-wise similarities of bait proteins, since the proposed algorithm uses the combined similarities to obtain seed clusters.

The pair-wise topological similarity among bait proteins is computed based on the number of commonly shared neighbours [17], which is generalized from the notion of Jaccard Similarity Coefficient [26].

a) Semantic similarity

The GO has three ontologies [20], MF, BP and CC, MF refers to information on what a gene product does. BP is related to a biological objective to which a gene product contributes. CC refers to the cellular location of the gene product, including cellular structures and complexes. The reader can refer to [20] for more details. In the paper, we use BP semantic similarity as the first instance.

The basic idea to calculate similarity between gene products is to calculate similarities between all terms that are used to annotate gene products. Let $b_{1}$ and $b_{2}$ be the two baits, and let $N\left(b_{1}\right)$ and $N\left(b_{2}\right)$ denote the set of neighbours of $b_{1}$ and $b_{2}$, respectively. The semantic similarity, *s_sim(b_1_,b_2_)*, has two numeric values, that is

(1) $$s\text{\_}sim\left(b_{1},b_{2}\right)=\left\{\begin{array}{cc} simValue, & if\;both\;b1\;and\;b2\;have\;annotations\\ -1, & if\;b1\;or\;b2\;doesn^{\prime }t\;have\;annotations \end{array}\right)$$

Here, *simValue *falls between [0,1], representing the closeness between pairs of proteins based on information derived from GO BP annotations. The value of -1 indicates that at least one of the two proteins has no annotations found. IEA ("Inferred from electronic annotation") annotations were excluded in the calculation due to their lack of reliability.

b) Combination of two similarities

The topology-based similarity and the semantic similarity are combined together to generate new pair-wise similarity measures for bait proteins. A simple way was adopted to combine the two different similarities as first trial by calculating the arithmetic average of topology-based similarities and semantic similarities.

(2) $$sim\left(b_{1},b_{2}\right)=\left\{\begin{array}{c} \frac{t\text{\_}sim\left(b_{1},b_{2}\right)+s\text{\_}sim\left(b_{1},b_{2}\right)}{2},\text{If}\;sim\left(b_{1},b_{2}\right)!=-1\\ t\text{\_}sim\left(b_{1},b_{2}\right),\text{If}\;s\text{\_}sim\left(b_{1},b_{2}\right)=-1 \end{array}\right)$$

Hereby, a network composed of similarities between pair-wise bait proteins could be obtained accordingly.

In the set of clusters obtained from expansion process, there exist overlap clusters. A merging process is applied to obtain the final set of clusters [25]. This paper is an extension from the conference paper [25], and details of BGCA algorithm can be referred to the study in [17].

## Results

### Preparation of data

In the study, two TAP-MS PPI networks are used. One is the dataset published by Gavin et al. [6] with 1993 bait proteins, 2671 prey proteins and 19157 bait-prey relationships; the other is the dataset published by Krogan et al. [8], which contains 2233 bait proteins, 5219 prey proteins and 40623 bait-prey relationships. There were 94 prey proteins which were suspected as non-specific contaminants [8], so they were excluded from the raw dataset used in Krogan et al. For convenience, the two datasets are named as Gavin_2006 and Krogan_2006 for short in this paper.

Two gold-standard datasets are employed in our experiments. One is obtained from the Munich database of Interacting Proteins (MIPS) [27], and the other is the set of hand-curated complexes derived from the Wodak lab CYC2008 catalogue [28]. The MIPS data file used is dated 18 May 2006 [27]. The MIPS category 550 was removed since it was defined by computerised algorithms only but contains no curated protein complexes [27]. As a result, the gold-standard data of MIPS contains 220 curated complexes. As for CYC2008 catalogue, 408 protein complexes are included.

### Evaluation strategy

In order to avoid biases in the evaluation of performance of proposed methods in the paper, the evaluation strategy is carefully designed and applied. The evaluation process in the paper is decided on the following:

1) A pre-process is applied on the gold-standard data and the set of predicted clusters. The similar pre-process was also adopted in several studies [16,29].

- For benchmark complexes in the gold-standard data, known complexes that contain proteins, all of which are not included in the network, are removed.

- For the set of candidate clusters, the clusters which have no overlaps with any benchmark complex are removed.

2) More than one quality measures are employed: precision/recall/FMeasure [29], sensitivity/Positive Predictive Value (PPV)/geometric accuracy [11], cluster-wise homogeneity/complex-wise homogeneity/geometric homogeneity [11], BH-Sensitivity/BH-Specificity/BH-FMeasure [10], and Jaccard FMeasure [29]. These quality measures calculate the degree of agreement between generated clusters obtained by clustering algorithms and well-studied protein complexes in a gold-standard set. The descriptions of these quality measures are provided in the section of quality measures.

3) Several typical clustering algorithms are employed to be compared with the proposed algorithm in this paper, including MCL [9,30], MCODE [10], CFinder [12], RRW [13], COACH [14], and CODEC [16]. For each algorithm, the clustering result to be evaluated was obtained by the optimal set of parameters.

4) The statistical significance of clustering results generated by the proposed algorithm is evaluated by computing quality scores of sets of randomly permutated complexes.

5) The robustness of the proposed algorithm to false positives and false negatives is evaluated by applying it to randomly altered networks.

### Pre-process of gold-standard datasets

The gold-standard datasets adopted in the study are MIPS [27] and CYC2008 [28]. As introduced in evaluation strategy, the gold-standard datasets will be pre-processed before being used in the evaluation. According to different PPI networks, proteins in each gold-standard that are not contained in the corresponding network are removed, and then the singleton complexes are excluded as well. Table 1 presents the statistics of number of proteins, number of complexes and average size of complexes in the original gold-standard datasets as well as in the gold-standard datasets being pre-processed which are used in the experiments.

**Table 1:** General statistics of two gold-standard datasets before and after pre-processing.

Gold-standard dataset	CYC2008	MIPS
**Original **		

No. of proteins	1627	1095

No. of complexes (size ≥ 2)	408	220

Ave. size of complexes	4.7	7.1

**On Gavin_2006 network**		

No. of proteins	1389	1041

No. of complexes (size ≥ 2)	360	205

Ave. size of complexes	5.5	8.1

**On Krogan_2006 network**		

No. of proteins	1592	1088

No. of complexes (size ≥ 2)	406	218

Ave. size of complexes	4.8	7.3

*Note the set of statistics under the "Original" header show the statistics of the original two gold-standard datasets before pre-processed; The set of statistics under the "On Gavin_2006 network" or " On Krogan_2006 network" header show the statistics of the two gold-standard datasets after being pre-processed on Gavin_2006 network or Krogan_2006 network, respectively.

### Selection of parameters

We select the parameters following a trial-and-error procedure. Unless indicated otherwise, the results reported in this paper were derived based on the following parameter settings: the hierarchical clustering was implemented with un-weighted average linkage and the cut-off values set to 0.3 and 0.25 for Gavin_2006 and Krogan_2006 networks, respectively. The overlapping rate is set to be 0.2.

In experiments, inflation of MCL is set as 3.0 in Gavin_2006 network and 2.0 in Krogan_2006 network respectively since results obtained accordingly are better comparing to other settings of inflation. For MCODE, on Gavin_2006, the depth equal is set to 100, node score percentage as 0.2, Haircut is TURE, Fluff is FALSE and the percentage for complex fluffing as 0.2; while on Krogan_2006, node score percentage is set as 0.1, and other parameters remain the same as those applied in Gavin_2006 network. For CFinder, the results generated from k=5 are employed since the results are better compared to other values of *k *based on quality measures. RRW has three parameters, restart probability, early cut-off and overlapping rate. The value of restart probability, early cut-off and overlapping rate are 0.6, 0.6, 0.2 for Gavin_2006 and 0.5, 0.7 and 0.2 for Krogan_2006, respectively. CODEC has two schemes, which are CODEC-w0 and CODEC-w1, and we compare our algorithm to both schemes of CODEC. We only use final predicted clusters from COACH, without considering its predicted core clusters.

### Experimental results and discussion

In order to gauge the effect after incorporating the semantic similarity in clustering process, we firstly compare proposed algorithm against the BGCA [17]. Since we use BP semantic similarity as the first instance, therefore, for convenience, the proposed algorithm is referred as BGCA+BP from now on. Then, we evaluate the performance of the proposed algorithm against several existing clustering methods. In the paper [26], it is presented that BGCA+BP performs better than BGCA in terms of six quality scores, such as sensitivity, PPV and geometric accuracy. In this paper, we use 9 more quality scores to further evaluate and compare the performance of BGCA+BP and BGCA.

A. The effect of incorporating semantic similarity on detecting protein complexes

Without incorporating semantic similarity, the similarities computed for pair-wise bait proteins are solely based on their locally topological feature, that is, the number of shared neighbours. In Tables 2 and 3, the evaluation results on predicted complexes generated by the BGCA and BGCA+BP from the Gavin_2006 and Krogan_2006 networks are presented.

**Table 2:** Evaluation of performance on Gavin_2006 and Krogan_2006 networks using MIPS gold-standard.

Network	Gavin_2006		Krogan_2006	
**Quality measures**	**BGCA**	**BGCA + BP**	**BGCA**	**BGCA + BP**

**Sensitivity**	0.357	**0.425**	0.257	**0.438**
**PPV**	0.601	**0.626**	0.455	**0.590**
**Geometric accuracy**	0.463	**0.516**	0.342	**0.509**

**Complex-wise Homogeneity**	0.324	**0.364**	0.156	**0.323**
**Cluster-wise Homogeneity**	0.692	**0.812**	0.642	**0.828**
**Geometric Homogeneity**	0.473	**0.544**	0.317	**0.517**

**Precision**	0.530	**0.614**	0.246	**0.449**
**Recall**	0.357	**0.425**	0.257	**0.438**
**PR value**	0.435	**0.511**	0.251	**0.444**

**Cluster-wise Jaccard**	0.387	**0.458**	0.159	**0.328**
**Complex-wise Jaccard**	0.267	**0.331**	0.149	**0.330**
**Jaccard FMeasure**	0.316	**0.384**	0.154	**0.329**

**BH-Specificity**	0.698	**0.837**	0.491	**0.824**
**BH-Sensitivity**	0.354	**0.425**	0.122	**0.361**
**BH-FMeasure**	0.470	**0.564**	0.195	**0.502**

Better measuring values are in bold.

**Table 3:** Evaluation of performance on Gavin_2006 and Krogan_2006 networks using CYC2008 gold-standard.

Network	Gavin_2006		Krogan_2006	
**Quality measures**	**BGCA**	**BGCA + BP**	**BGCA**	**BGCA + BP**

**Sensitivity**	0.461	**0.480**	0.300	**0.419**
**PPV**	**0.711**	0.709	0.550	**0.595**
**Geometric accuracy**	0.573	**0.583**	0.406	**0.499**

**Complex-wise Homogeneity**	0.307	**0.326**	0.134	**0.221**
**Cluster-wise Homogeneity**	0.819	**0.896**	0.745	**0.846**
**Geometric Homogeneity**	0.502	**0.540**	0.316	**0.432**

**Precision**	0.670	**0.714**	0.371	**0.428**
**Recall**	0.461	**0.480**	0.300	**0.419**
**PR value**	0.556	**0.585**	0.334	**0.423**

**Cluster-wise Jaccard**	0.536	**0.583**	0.255	**0.321**
**Complex-wise Jaccard**	0.361	**0.380**	0.204	**0.293**
**Jaccard FMeasure**	0.432	**0.460**	0.227	**0.306**

**BH-Specificity**	0.867	**0.901**	0.740	**0.840**
**BH-Sensitivity**	0.340	**0.352**	0.136	**0.232**
**BH-FMeasure**	0.489	**0.506**	0.229	**0.363**

Better measuring values are in bold.

In [26], BGCA+BP demonstrated higher accuracy and homogeneity value than BGCA, which can also be seen in the Tables 2 and 3. In terms of other 9 quality measures, it can be seen that the BGCA+BP also obtained better values. For example, in Table 2, BGCA+BP achieves 20% increase in BH-FMeasure on Gavin_2006 network, while the BH-FMeasure value that BGCA+BP obtained is achieves one and a half time as much as that of BGCA. The similar observation can be obtained in Table 3. The fact that BGCA+BP consistently achieves better scores according to the quality measures than BGCA indicates that, combination of topological similarity and semantic similarity can enhance the accuracy of predicting protein complexes.

B. Comparison to other clustering methods

Table 4 presents some statistics, the number and the average size, of clusters generated by all algorithms on the two TAP-MS networks. On both networks, the COACH tends to generate clusters of the largest average size, while RRW has clusters of the smallest average size. The CODEC yields the largest number of clusters.

**Table 4:** Number and average size of generated clusters from different methods on Gavin_2006 network and Krogan_2006 network.

	MCL	MCODE	CFinder	RRW	COACH	CODEC-w0	CODEC-w1	BGCA+BP
Gavin_2006								

No. of clusters	782	100	65	474	612	1082	1005	542
Ave. size	5.4	12.1	16.4	2.1	78.1	17.3	13.8	5.0

Krogan_2006								

No. of clusters	1548	73	73	690	1927	8348	2973	511
Ave. size	5.5	25.2	15.1	2.1	181.8	16.1	16.2	5.3

• Analysis of experimental results on Gavin_2006 network

Table 5 and Table 6 present quality scores of clustering results generated by different clustering algorithms from Gavin_2006 networks, compared with gold-standards of MIPS and CYC2008.

**Table 5:** Evaluation results on Gavin_2006 network using MIPS gold-standard.

Quality measures	BGCA+BP	MCL	MCODE	CFinder	RRW	COACH	CODEC-w0	CODEC-w1
**Sensitivity**	0.425	0.413	0.271	0.334	0.107	**0.484**	0.451	0.450
**PPV**	**0.626**	0.492	0.332	0.330	0.500	0.140	0.486	0.556
**Geometric accuracy**	**0.516**	0.451	0.300	0.332	0.232	0.261	0.468	0.500

**Complex-wise Homogeneity**	**0.364**	0.279	0.138	0.109	0.104	0.061	0.250	0.273
**Cluster-wise Homogeneity**	0.812	0.656	0.601	0.532	**0.927**	0.024	0.060	0.074
**Geometric Homogeneity**	**0.544**	0.428	0.288	0.241	0.311	0.038	0.122	0.142

**Precision**	0.614	0.334	0.212	0.251	**0.848**	0.058	0.296	0.400
**Recall**	0.425	0.413	0.271	0.334	0.107	**0.484**	0.451	0.450
**PR value**	**0.511**	0.372	0.239	0.289	0.302	0.167	0.365	0.424

**Cluster-wise Jaccard**	0.458	0.245	0.146	0.185	**0.543**	0.042	0.140	0.178
**Complex-wise Jaccard**	**0.331**	0.242	0.115	0.128	0.097	0.231	0.289	0.295
**Jaccard FMeasure**	**0.384**	0.244	0.129	0.152	0.165	0.071	0.188	0.222
**BH-Specificity**	**0.837**	0.655	0.404	0.619	0.739	0.141	0.213	0.273
**BH-Sensitivity**	0.425	0.302	0.094	0.130	0.087	0.338	0.610	**0.658**
**BH-FMeasure**	**0.564**	0.413	0.152	0.215	0.156	0.199	0.316	0.386

Better measuring values are in bold.

**Table 6:** Evaluation results on Gavin_2006 network using CYC2008 gold-standard.

Quality measures	BGCA+BP	MCL	MCODE	CFinder	RRW	COACH	CODEC-w0	CODEC-w1
**Sensitivity**	0.480	0.538	0.338	0.390	0.089	0**.596**	0.584	0.582
**PPV**	0.709	0.571	0.342	0.365	**0.764**	0.120	0.511	0.546
**Geometric accuracy**	**0.583**	0.555	0.340	0.377	0.261	0.268	0.546	0.564

**Complex-wise Homogeneity**	**0.326**	0.295	0.123	0.087	0.088	0.048	0.234	0.272
**Cluster-wise Homogeneity**	0.896	0.816	0.748	0.613	**0.989**	0.030	0.086	0.107
**Geometric Homogeneity**	**0.540**	0.490	0.303	0.231	0.295	0.038	0.141	0.171

**Precision**	0.714	0.419	0.268	0.324	**0.891**	0.066	0.311	0.426
**Recall**	0.480	0.538	0.338	0.390	0.089	**0.596**	0.584	0.582
**PR value**	**0.585**	0.475	0.301	0.356	0.281	0.198	0.426	0.498

**Cluster-wise Jaccard**	0.583	0.335	0.208	0.260	**0.633**	0.053	0.169	0.230
**Complex-wise Jaccard**	0.380	0.319	0.146	0.139	0.082	0.272	0.362	**0.383**
**Jaccard FMeasure**	**0.460**	0.326	0.172	0.181	0.145	0.088	0.230	0.287

**BH-Specificity**	**0.901**	0.815	0.610	0.804	0.875	0.252	0.305	0.459
**BH-Sensitivity**	0.352	0.315	0.103	0.115	0.079	0.360	0.578	**0.691**
**BH-FMeasure**	0.506	0.454	0.176	0.201	0.144	0.297	0.399	**0.552**

Better measuring values are in bold.

Based on figures in Table 5, COACH has the highest sensitivity value. The proposed BGCA+BP algorithm achieves the best PPV, as well as best geometric accuracy. The sensitivity value indicates the average fraction of proteins inside a known complex, which is correctly grouped together in the generated clustering result. A large cluster size can artificially increase the sensitivity value, since a large cluster may contain proteins which belong to more than one complex. Small size of cluster may also increase PPV. The high sensitivity value, but low PPV value, of COACH indicates that the high sensitivity value results from large sized clusters generated by COACH. Meanwhile, the high PPV value but the poor sensitivity value of RRW demonstrates that very few benchmark complexes are uncovered in the results generated by RRW.

Apart from COACH and RRW, the scores of sensitivity and PPV obtained by the rest the algorithms are quite balanced. The BGCA and MCL have higher sensitivity than MCODE and CFinder, since more benchmark complexes are uncovered according to the number of matched complexes. The best geometric accuracy suggests that the BGCA+BP can achieve a much better performance as the value of the accuracy reflects the general performance of a clustering algorithm based on the estimation of the overall correspondence between the set of generated clusters and the set of gold-standard complexes. When compared with CYC2008 gold-standard, a similar observation can be obtained, as shown in Table 6.

Homogeneity is the product of the fraction of members in a cluster found in an annotated complex by the fraction of members in the complex found in a cluster. High homogeneity indicates a bi-directional correspondence between a cluster and a complex. The maximal value of homogeneity is 1 when a cluster matches perfectly with a complex, which means that the cluster consists of all its members identified in the complex. As shown in Tables 5, and 6, the BGCA+BP achieves the best performance in terms of the geometric homogeneity value, which reflects the general agreement between identified clusters and benchmark complexes, as well as the quality of a clustering result as a whole.

The precision value of a predicted cluster calculates the absolute fraction of proteins within a cluster which are also found in a benchmark complex. The clustering-wise precision value represents the average precision values over all clusters. RRW has the highest precision score, but again very poor recall value, therefore, the overall PR value for RRW is low, regardless which gold-standard datasets are used. On the other hand, COACH obtains the highest recall value but very low precision. Again, overall, the BGCA+BP achieves the best PR value.

Jaccard index measures the impact of overlapped sections on both predicted clusters and the corresponding benchmark complex, since it considers the proportion of overlap size in the union set of a predicted cluster and a benchmark complex. High Jaccard index suggests that the set of clustering results is very well matched to the set of benchmark complexes. The second best cluster-wise Jaccard index, the best complex-wise Jaccard index and also the best FMeasure obtained by the proposed method, suggest that the set of clustering results of the proposed method is better matched to the set of benchmark complexes included in all three gold-standards than other algorithms.

The BH-Sensitivity is used to measure the percentage of benchmark complexes recovered by generated clusters whose overlap score satisfies the given threshold. The BH-Specificity value measures fraction of generated clusters that match benchmark complexes. On Gavin_2006 network, observed from Tables 5 and 6, compared with the two gold-standards separately, BGCA+BP obtains the highest value in BH-Specificity, while CODEC-w1 has the best BH-Sensitivity. However, when compared with MIPS gold-standard, BGCA+BP has best value in BH-FMeasure; while compared with CYC2008, CODEC-w1 achieves better BH-FMeasure. The reason may be due to the incompleteness of each gold-standard.

By achieving best value in most quality measures, it can be concluded that BGCA+BP outperforms other algorithms on Gavin_2006 network.

• Analysis of experimental results on Krogan_2006 network

Tables 7 and 8 present the quality scores for clustering results produced from Krogan_2006 network.

**Table 7:** Evaluation results on Krogan_2006 network using MIPS gold-standard.

Quality measures	BGCA+BP	MCL	MCODE	CFinder	RRW	COACH	CODEC-w0	CODEC-w1
**Sensitivity**	0.438	0.183	0.219	0.290	0.028	**0.564**	0.420	0.404
**PPV**	**0.590**	0.565	0.152	0.342	0.558	0.094	0.386	0.362
**Geometric accuracy**	**0.509**	0.322	0.182	0.315	0.124	0.231	0.403	0.382

**Complex-wise Homogeneity**	**0.323**	0.202	0.049	0.078	0.037	0.019	0.226	0.215
**Cluster-wise Homogeneity**	0.828	0.587	0.409	0.656	**1.000**	0.002	0.015	0.032
**Geometric Homogeneity**	**0.517**	0.344	0.141	0.226	0.192	0.007	0.059	0.083

**Precision**	0.449	0.355	0.069	0.237	**0.750**	0.040	0.180	0.175
**Recall**	0.438	0.183	0.219	0.290	0.028	**0.564**	0.420	0.404
**PR value**	**0.444**	0.255	0.123	0.262	0.144	0.150	0.275	0.266

**Cluster-wise Jaccard**	0.328	0.203	0.055	0.206	**0.528**	0.034	0.105	0.115
**Complex-wise Jaccard**	**0.330**	0.125	0.049	0.103	0.026	0.162	0.250	0.241
**Jaccard FMeasure**	**0.329**	0.154	0.051	0.137	0.049	0.056	0.148	0.155

**BH-Specificity**	0.824	0.280	0.154	0.808	**0.875**	0.024	0.212	0.337
**BH-Sensitivity**	0.361	0.098	0.018	0.100	0.032	0.190	**0.845**	0.781
**BH-FMeasure**	**0.502**	0.145	0.033	0.179	0.062	0.043	0.338	0.470

Better measuring values are in bold.

**Table 8:** Evaluation results on Krogan_2006 network using CYC2008 gold-standard.

Quality measures	BGCA+BP	MCL	MCODE	CFinder	RRW	COACH	CODEC-w0	CODEC-w1
**Sensitivity**	0.419	0.269	0.275	0.346	0.036	**0.660**	0.595	0.562
**PPV**	0.595	0.653	0.135	0.389	**0.739**	0.076	0.399	0.422
**Geometric accuracy**	**0.499**	0.419	0.193	0.366	0.163	0.224	0.487	0.487

**Complex-wise Homogeneity**	0.221	**0.242**	0.036	0.063	0.042	0.015	0.232	0.218
**Cluster-wise Homogeneity**	0.846	0.706	0.474	0.566	**1.000**	0.003	0.024	0.048
**Geometric Homogeneity**	**0.432**	0.413	0.131	0.189	0.205	0.007	0.075	0.102

**Precision**	0.428	0.406	0.080	0.352	**0.797**	0.040	0.206	0.238
**Recall**	0.419	0.269	0.275	0.346	0.036	**0.660**	0.595	0.562
**PR value**	**0.423**	0.331	0.148	0.349	0.169	0.162	0.350	0.366

**Cluster-wise Jaccard**	0.321	0.259	0.065	0.304	**0.598**	0.035	0.138	0.170
**Complex-wise Jaccard**	0.293	0.171	0.054	0.142	0.029	0.194	**0.344**	0.323
**Jaccard FMeasure**	**0.306**	0.206	0.059	0.194	0.055	0.059	0.197	0.223

**BH-Specificity**	0.840	0.345	0.226	0.800	**0.941**	0.047	0.324	0.502
**BH-Sensitivity**	0.232	0.119	0.017	0.090	0.040	0.196	**0.857**	0.790
**BH-FMeasure**	0.363	0.177	0.032	0.162	0.076	0.075	0.470	**0.614**

Better measuring values are in bold.

Similar to results of Gavin_2006 network, the BGCA+BP achieves best value in most of overall quality measures, such as geometric accuracy, geometric homogeneity, PR value, and Jaccard FMmeasure. Again, as for BH-FMeasure, BGCA+BP has best value when using MIPS as gold-standard, whereas CODEC-w1 is the best when comparing with CYC2008 gold-standard.

Though the BGCA+BP does not have all the best values, it still achieves most of them, which indicates that BGCA+BP outperforms other clustering algorithms in terms of the overall performance measurement.

C. Statistical significance of clustering results

This section investigates the estimates of random expectation of correct grouping by randomising predicted complexes sets. A set of predicted complexes from original networks are randomised by shuffling nodes between different complexes while keeping the number of complexes, and the sizes of corresponding complexes, unchanged. The resulting set of permuted clusters is then evaluated by quality measures using gold-standards. If quality scores of original set of generated clusters are close to those of the random set, it indicates that the corresponding clustering algorithm yields a set of predicted complexes which is not significantly better than a randomly generated set of complexes.

The process of creating permuted clusters is as follows. The original set of generated clusters was concatenated into a list of proteins. Then the Fisher-Yates shuffle [31,32] was applied to the list of proteins. The procedure of shuffling was repeated 1,000 times, and then the list was divided into groups in a way that preserves the sizes of original complexes and the number of complexes. This grouping was then evaluated by each quality measure. Since the Fisher-Yates shuffle chooses any possible permutation of a list with equal probability, the resulting set of permuted clusters can be used to obtain an unbiased estimate for the expected value of any chosen quality score.

The permutation process was repeated 1,000 times, resulting in 1,000 clustering sets. Each clustering set was evaluated by those quality scores and the average score corresponding to each metric was calculated. The *p*-value is obtained by calculating the number of times that a randomised set of clustering results had a higher value in quality scores than that of the original clustering set, divided by the total number of permutations, which is 1,000 here. If *p*-value is less than 0.05, it indicates that the high performance achieved by the proposed algorithm is unlikely to occur by chance. In this study, we use the Bonferonni correction to counteract the problem of multiple comparisons [33].

Without loss of generality, we only use one gold-standard dataset, CYC2008. Table 9 displays the expected values of BGCA+BP on Gavin_2006 network and Krogan_2006 network using CYC2008 gold-standard, respectively. The quality scores employed to measure the effect of randomised clusters include the fraction of matched complexes, geometric accuracy, geometric homogeneity, PR values, Jaccard FMeasure and BH-FMeasure.

**Table 9:** Expected values of evaluation results of randomised clustering of BGCA+BP on Gavin_2006 and Krogan_2006 networks using CYC2008 gold-standard.

	Gavin_2006			Krogan_2006		
**Quality measures**	Original	Random average	*p*-value	Original	Random average	*p*-value

**Geometric accuracy**	0.583	0.095	0.000	0.499	0.095	0.000

**Geometric Homogeneity**	0.540	0.123	0.000	0.432	0.093	0.000

**PR value**	0.585	0.072	0.000	0.423	0.064	0.000

**Jaccard FMeausre**	0.460	0.023	0.000	0.306	0.017	0.000

**BH-Fmeasure**	0.506	0.001	0.000	0.363	0.001	0.000

*p-value is corrected.

It can be observed that the average quality scores in case of Jaccard FMeasure and BH-FMeasure are close to zero. Though the values of geometric accuracy, geometric homogeneity, and PR value are higher, they are still very small, compared with those of the original set. Very low *p*-values indicate that the original set of clusters is significantly better than the randomised clustering sets.

D. Robustness of the proposed algorithm

In order to evaluate the robustness of the proposed algorithms to false positives and false negatives, various levels of alteration have been made by adding or deleting percentages of edges with respect to the number of edges in the original Gavin_2006 network. The strategy of altering graph in [11] is adopted in the study. Increasing fraction of edges (0%, 5%, 10%, 20%, 40%, 80%, 100%) are randomly added to the original graph. Similarly, increasing fraction of edges (0%, 5%, 10%, 20%, 40%, 80%) are randomly deleted from the original network. Specifically, the proportion of edges which are added or removed is obtained based on the number of edges in the original graph. Take the Gavin_2006 network as an example, 5% edges are equal to 964 edges (5% of 19,277 edges). In the experiment, the Network Analysis Tools (NeAT) [34] has been applied to alter the network. Note, in the alteration of graphs applied in the study, self-loops and duplicated edges are not allowed.

**Figure 1:**
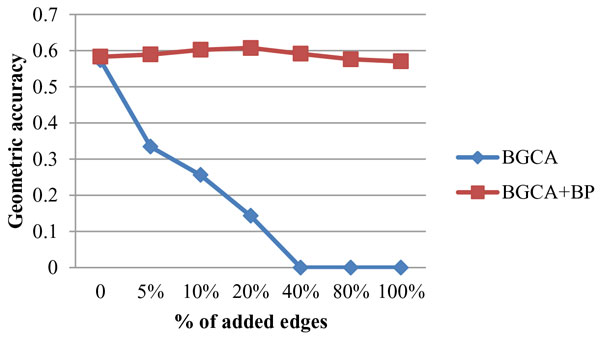
**Robustness of BGCA and BGCA+BP: impact of edge addition on geometric accuracy**.

**Figure 2:**
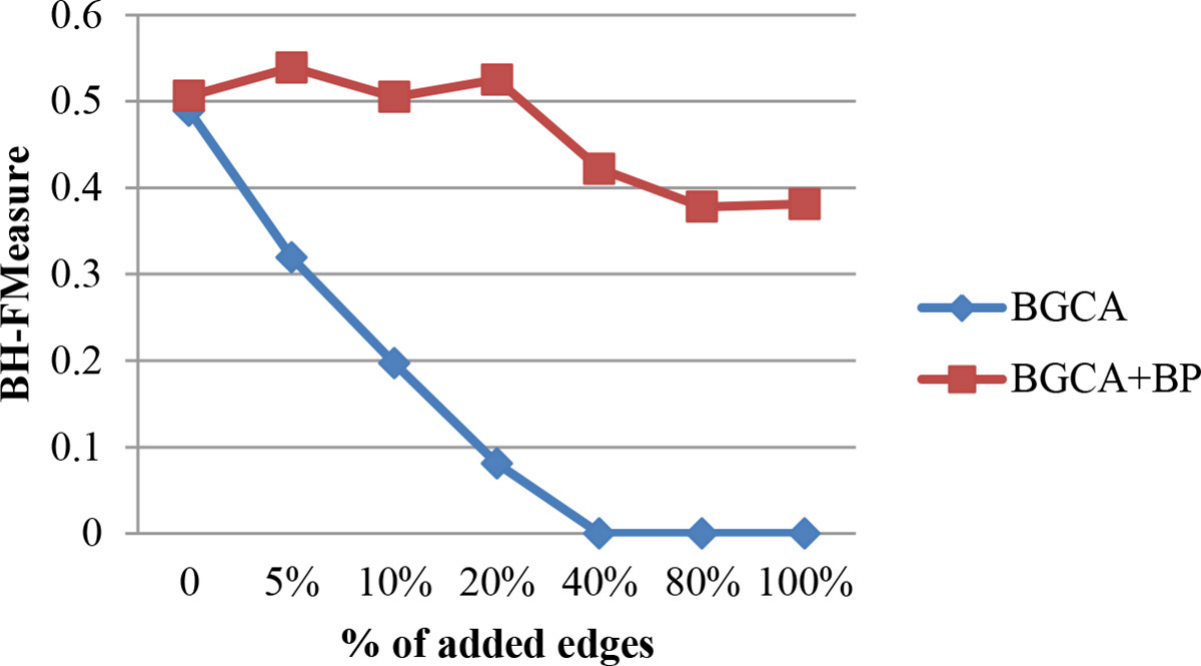
**Robustness of BGCA and BGCA+BP: impact of edge addition on BH-FMeasure**.

In order to demonstrate the advantage of incorporating semantic similarity, in the experiment, the performance of BGCA in the context of detecting protein complexes from randomly altered graphs is also presented. Geometric accuracy and BH-FMeasure were used to demonstrate the impact on clustering results of BGCA by introducing noises into the network. Figure 1 and Figure 2 present the impact on geometric accuracy and BH-FMeasure of the BGCA and BGCA+BP, when edges were randomly added.

Observation can be made from Figure 1, as for BGCA+BP, the curve representing the geometric accuracy is smooth. The geometric accuracy increases slightly first since 5% edges were added, and the highest value is obtained when 40% edges were added. The geometric accuracy starts to decline when more than 40% edges were added. However, the change in geometric accuracy is still trivial even when 100% edges were added compared to that in the original graph. The curve represented that the BH-FMeasure fluctuates slightly in the interval when edges were added increasingly from 5% to 20%. The best value is obtained when 5% edges were added and then the BH-FMeasure drops and rises again when 20% edges were added. When more than 20% edges were added, the BH-FMeasure declines greatly but the curve becomes smooth after 80% and 100% edges were added. With regard to BGCA, the curve representing geometric accuracy of the BGCA drops drastically as 5% edges were randomly added to the original graph. When adding 40% edges, the value of geometric accuracy of the BGCA falls down to 0, since there are no generated clusters which match to any benchmark complexes. Similar observations can be obtained from Figure 2. With semantic similarity, the BGCA+BP demonstrate much more robustnes than the BGCA in the case of randomly adding edges to the original graph.

**Figure 3:**
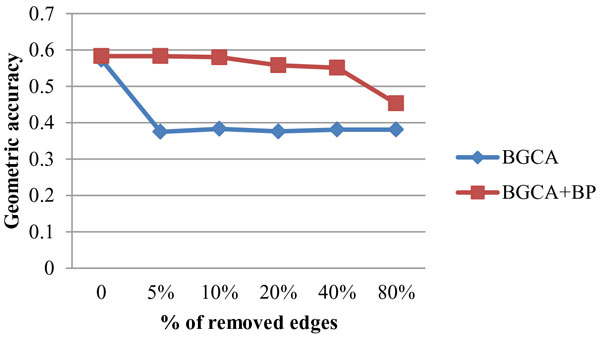
**Robustness of BGCA and BGCA+BP: impact of edge deletion on geometric accuracy**.

**Figure 4:**
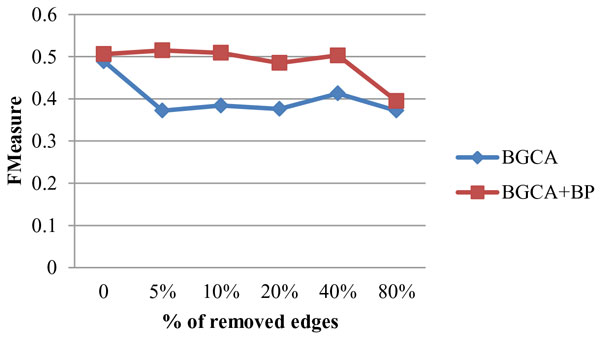
**Robustness of BGCA and BGCA+BP: impact of edge deletion on BH-FMeasure**.

Figure 3 and Figure 4 present the impact on geometric accuracy and BH-FMeasure when randomly deleting edges from the original graph. The geometric accuracy of BGCA+BP is affected slightly until more than 40% edges were deleted from the original graph. The BH-FMeasure also drops when removing 40% edges from the original graph. The value of geometric accuracy and BH-FMeasure of the BGCA+BP only drops when more than 40% edges are removed. It shows that the BGCA+BP is also robust to edge deletion. As for BGCA, the trend of curves representing both geometric accuracy and BH-FMeasure is similar. The curves keep almost unchanged after a drop when the fraction of deleted edges is increased from 0% to 5%, demonstrating the BGCA is relatively robust in the case of edge deletion, compared with that of edge addition.

From these observations, it can be concluded that by incorporating semantic similarity, the proposed algorithm is quite robust to the noises in PPI networks.

## Conclusions

In this paper, we propose a new algorithm combining topological features and semantic similarities between proteins to discover protein complexes in TAP-MS PPI networks. The proposed algorithm is extended from a previously proposed algorithm, i.e. BGCA [17]. It has been tested on two published TAP-MS PPI networks, Gavin_2006 network and Krogan_2006 network. The proposed algorithm inherits the main feature of BGCA which is that it detects protein complexes by taking co-complex relations into account from TAP-MS data. Results indicate that by integrating GO-driven similarity knowledge into clustering process, the proposed algorithm outperforms BGCA as well as several state-of-art clustering techniques. Not only a higher accuracy has been achieved, the proposed algorithm also significantly improves the robustness of BGCA to the noise inherent in protein interaction data generated by TAP-MS.

In this paper, the strategy of combining topological similarity and semantic similarity in BGCA is developed by calculating the average value, in which the weights assigned to semantic similarity and topological similarity are the same. The behaviour of the algorithm by using other weighting schemes deserves further investigation. Moreover, incorporating other types similarity information, such as those derived from CC and MF ontologies [20] into the algorithm for further improvement will be considered as well.

## Quality measures

This section introduces quality measures that have been used in the study. These quality measures calculate the degree of agreement between predicted clusters obtained by clustering algorithms and well-studied clusters in a reference set. In application to identify complexes in PPI networks, the reference set can be built from gold-standard databases, such as CYC2008 [28] and MIPS [27]. Generally, the value of these quality measures falls into the interval between 0 and 1. The higher the value, the better quality of clustering and better performance a clustering algorithm has.

Let  C be the set of predicted clusters and  M be the set of benchmark protein complexes. Let  n be the number of clusters in  C, and  m be the number of complexes, then a n×m confusion matrix  Z is constructed for comparison between predicted clusters and gold-standard complexes. The $i^{th}$ row corresponds to candidate cluster  i while the $j^{th}$ column stands for benchmark complex  j. The entry $z_{ij}$ represents size of intersection between $i^{th}$ row and $j^{th}$ column, which is the number of proteins which are identified as members in cluster  i and also belongs to complex  j as well. $z_{i}$ is the size of $i^{th}$ cluster while $z_{j}$ represents size of $j^{th}$ complex.

• **Sensitivity, Positive Predictive Value (PPV), and Geometric Accuracy**

Geometric accuracy, which was proposed by Brohée and Helden [11], measures degree of correspondence between the set of predicted clusters and the set of benchmark complexes. Geometric accuracy contains two other parameters, sensitivity and PPV.

Sensitivity is defined as the proportion of proteins of benchmark complex  j which are identified in the predicted cluster  i. The general sensitivity is obtained by the weighted average of maximal *sensitivity *of each complex over all complexes

(3) $$Sensitivity=\left(\sum_{j=1}^{m}z_{j}max_{i=1}^{n}\left(z_{ij}/z_{j}\right)\right)/\sum_{j=1}^{m}z_{j}$$

PPV represents the maximal fraction of a predicted cluster  i belongs to the same benchmark complex. It indicates the reliability with which predicted cluster  i predicts that a protein belongs to its best-matching benchmark complex. $\overset{m}{\underset{j=1}{\sum }}z_{ij}$ is the marginal sum of the predicted cluster  i.

(4) $$PPV=\left(\sum_{i=1}^{n}z_{i}\text{max}\left(z_{ij}/\sum_{j=1}^{m}z_{ij}\right)\right)/\sum_{i=1}^{n}\sum_{j=1}^{m}z_{ij}$$

Geometry accuracy is defined as the geometric mean of the product general sensitivity and PPV,

(5) $$Geometric\;accuracy=\sqrt{Sensitivity\times PPV}$$

Accuracy reflects the trade-off between sensitivity and PPV. A high accuracy value requires a high performance for both measures. The higher the accuracy values the better quality of a clustering result.

• **Homogeneity**

Homogeneity [35], called separation by Brohée and Helden [11], provides a measure of degree of bidirectional correspondence between a predicted cluster and a benchmark complex. It is the product of the fraction of proteins found in a cluster by the fraction of proteins annotated in the complex, relative to the marginal sum of the row or the column.

The cluster-wise homogeneity $hM_{cl_{i}}$ is defined to represent the frequency of distribution of proteins detected as members in the same cluster  i over annotated complexes. The cluster-wise homogeneity $hM_{cl_{i}}$ calculates the sum of the homogeneity value for a cluster  i,

(6) $$hM_{cl_{i}}=\overset{m}{\underset{j=1}{\sum }}hM_{ij}$$

Similarly, Complex-wise homogeneity $hM_{co_{j}}$ shows the frequency of the fraction of proteins in a same benchmark complex  j over all the predicted clusters. The complex-wise homogeneity $hM_{co_{j}}$ is calculated as the sum of homogeneity value for a benchmark complex, that is,

(7) $$hM_{co_{j}}=\overset{n}{\underset{i=1}{\sum }}hM_{ij}$$

To measure the general cluster-wise homogeneity $hM_{cl}$ and complex-wise homogeneity $hM_{co}$, the average values of $hM_{cl_{i}}$ and $hM_{co_{j}}$ over all predicted clusters and benchmark complexes are calculated, respectively.

(8) $$hM_{cl}=\frac{\sum_{i=1}^{n}hM_{cl_{i}}}{n}$$

(9) $$hM_{co}=\frac{\sum_{j=1}^{m}hM_{co_{j}}}{m}$$

To estimate general homogeneity over a clustering, the general homogeneity hM is defined as the geometric mean of the product of general cluster-wise homogeneity and complex-wise homogeneity.

(10) $$Homogeneity=\sqrt{hM_{cl}\times hM_{co}}$$

Homogeneity reflects relative ratio of distribution of overlapping intersections between annotated complexes and generated clusters. When proteins are allowed to be assigned to multiple clusters, the value cluster-wise homogeneity will be lower and thus the general homogeneity value will be lower.

• **Precision, Recall and PR-value**

In a clustering task, the precision is defined as the fraction of True Positives (TPs) which are correctly labelled items in the predicted class, and recall is the fraction of TPs in a reference class [29]. In the context of detection of protein complexes in PPI networks, precision of cluster  i is the number of TPs divided by the size of this cluster while recall of complex  j is the number of TPs divided by the size of the benchmark complex [29]. Here, TPs are proteins found in the predicted cluster and also annotated in the benchmark complex [29]. The number of TPs between cluster  i and complex  j is equal to the size of intersection in the confusion table defined as above. Thus, precision $Pr_{ij}$ and recall $Re_{ij}$ of cluster  i and complex  j are computed as follows:

(11) $$Pr_{ij}=\frac{z_{ij}}{z_{i}}$$

(12) $$Re_{ij}=\frac{z_{ij}}{z_{j}}$$

where $z_{i}$ and $z_{j}$ represents size of predicted cluster  i and size of benchmark complex  j, respectively. The maximal precision value for cluster  i over all benchmark complexes is used as precision of the predicted cluster  i.

(13) $$Pr_{cl_{i}}=max_{j}^{m}Pr_{ij}$$

The recall for the benchmark complex  j is defined as:

(14) $$Re_{co_{j}}=max_{i}^{n}Re_{ij}$$

Recall reveals how well a benchmark complex is covered by the corresponding cluster. Precision here is obtained by dividing the size of the local cluster, measuring percentage of TPs in the local cluster.

A general precision is obtained by calculating the weighted average of precision over all predicted clusters.

(15) $$precision=\frac{\sum_{i=1}^{n}z_{i}\cdot Pr_{cl_{i}}}{\sum_{i=1}^{n}z_{i}}$$

The general recall also uses the weighted average of recall values over all benchmark complexes,

(16) $$recall=\frac{\sum_{j=1}^{m}z_{j}\cdot Re_{co_{j}}}{\sum_{j=1}^{m}z_{j}}$$

PR value is the harmonic mean of precision and recall, used to reflect the degree of TPs predicted in a clustering as well as general correspondence between predicted clusters and benchmark complexes.

(17) $$PR\;value=\frac{2\times precision\times recall}{precision+recall}$$

• **BH-Sensitivity and BH-specificity**

A different definition of sensitivity from the one which was proposed by Brohée and Helden [11] was used by Bader and Hogue [10]. In order to differentiate the sensitivity used by Broheé and Helden [11], the sensitivity and specificity introduced in this section are referred as BH-Sensitivity and BH-Specificity, where BH is the initials of the authors, Bader and Hogue [10]. In the set of predicted clusters, the numbers of TPs, True Negatives (TN), FPs and FNs depend on how threshold is selected relative to sets of gold-standard complexes. An overlap score  w was proposed to measure how significantly a predicted cluster matches a benchmark complex by Bader and Hogue in 2003 [10].

(18) $$w=\frac{{\left(z_{ij}\right)}^{2}}{z_{i}\times z_{j}}$$

Where $z_{ij}$ represents the number of overlapping proteins between the predicted cluster  i and the benchmark complex  j, $z_{i}$ is the size of predicted cluster  i and $z_{j}$ is the size of the benchmark complex  j.

The number of TP is defined as the number of predicted clusters with  w over a threshold value and the number of FP is the total number of predicted clusters minus TP. The number of FN is defined as the number of benchmark complexes that are not matched by predicted clusters, while the number of TN is the number of benchmark complexes that are matched by predicted clusters with  w over a threshold value. The formula used to calculate sensitivity and specificity are presented below:

(19) $$BH-Sensitivity=\frac{TP}{TP+FN}$$

(20) $$BH-Specificity=\frac{TP}{TP+FP}$$

In this study, the threshold value of  w is set to 0.2. The *f*-measure value of BH-sensitivity and BH-specificity is also employed to measure the overall performance of a clustering algorithm.

(21) $$BH-Fmeasure=\frac{2\times BH-Sensitivity\times BH-Specificity}{BH-Sensitivity+BH-Specificity}$$

• **Jaccard index**

Extended from Jaccard similarity measure [26], Jaccard index calculates the fraction of intersection between a predicted cluster and a benchmark complex over the union set of the cluster and benchmark complex [29].

In order to measure how well the group of predicted clusters map to benchmark complexes, for each cluster  i, the benchmark complex  j that maximises overlap between itself and the cluster  i is found, that is,

(22) $$Jac_{cl_{i}}=max_{j=1}^{m}\frac{z_{ij}}{\left|z_{i}\cup ,z_{j}\right|}$$

Where $\left|z_{i}\cup z_{j}\right|$ represents the size of the union set of predicted cluster  i and benchmark complex  j. Then, a weight average of cluster-wise Jaccard index is calculated over all predicted clusters, that is,

(23) $$Jac_{cl}=\frac{\sum_{i=1}^{n}z_{i}\cdot Jac_{cl_{i}}}{\sum_{i=1}^{n}z_{i}}$$

Similarly, as to measure how well a set of benchmark complexes correspond to the set of predicted clusters, a complex-wise Jaccard index is calculated. First, for each benchmark complex  j, a maximum Jaccard index is obtained by

(24) $$Jac_{co_{j}}=max_{i=1}^{n}\frac{z_{ij}}{\left|z_{i}\cup z_{j}\right|}$$

Then, the complex-wise Jaccard index over the set of benchmark complexes is calculated,

(25) $$Jac_{co}=\frac{\sum_{j=1}^{m}z_{j}\cdot Jac_{co_{j}}}{\sum_{j=1}^{m}z_{j}}$$

Finally, the general Jaccard index is defined as the harmonic mean of $Jac_{cl}$ and $Jac_{co}$, that is:

(26) $$Jaccard\;Fmeasure=\frac{2\times Jac_{cl}\times Jac_{co}}{Jac_{cl}+Jac_{co}}$$

Jaccard measure reflects the degree of bi-directional correspondence between the set of predicted clusters and the group of benchmark complexes. Higher Jaccard measure value indicates that predicted clusters very well match to the group of benchmark complexes and vice versa.

## Competing interests

The authors declare that they have no competing interests.

## Authors' contributions

BC contributed to algorithms design and carried out all programming and analyses as a Ph.D student in the University of Ulster. HYW, HZ and HW supervised this study, guided algorithms development, data analysis and contributed to the preparation of this manuscript. All authors read and approved the final manuscript.

## Declaration

The publication costs for the article will be funded by the Computer Science Research Institute, University of Ulster.

This article has been published as part of *Proteome Science *Volume 11 Supplement 1, 2013: Selected articles from the IEEE International Conference on Bioinformatics and Biomedicine 2012: Proteome Science. The full contents of the supplement are available online at http://www.proteomesci.com/supplements/11/S1.
